# Recent Advances in mmWave-Radar-Based Sensing, Its Applications, and Machine Learning Techniques: A Review

**DOI:** 10.3390/s23218901

**Published:** 2023-11-01

**Authors:** A. Soumya, C. Krishna Mohan, Linga Reddy Cenkeramaddi

**Affiliations:** 1 Department of Computer Science Engineering, Indian Institute of Technology, Hyderabad 502285, India; cs21resch15003@iith.ac.in (A.S.); ckm@cse.iith.ac.in (C.K.M.); 2 Department of Information and Communication Technology, University of Agder, 4879 Grimstad, Norway

**Keywords:** mmWave radar, mmWave radar applications, machine learning, industrial applications, medical applications, automotive applications, military applications, computer vision

## Abstract

Human gesture detection, obstacle detection, collision avoidance, parking aids, automotive driving, medical, meteorological, industrial, agriculture, defense, space, and other relevant fields have all benefited from recent advancements in mmWave radar sensor technology. A mmWave radar has several advantages that set it apart from other types of sensors. A mmWave radar can operate in bright, dazzling, or no-light conditions. A mmWave radar has better antenna miniaturization than other traditional radars, and it has better range resolution. However, as more data sets have been made available, there has been a significant increase in the potential for incorporating radar data into different machine learning methods for various applications. This review focuses on key performance metrics in mmWave-radar-based sensing, detailed applications, and machine learning techniques used with mmWave radar for a variety of tasks. This article starts out with a discussion of the various working bands of mmWave radars, then moves on to various types of mmWave radars and their key specifications, mmWave radar data interpretation, vast applications in various domains, and, in the end, a discussion of machine learning algorithms applied with radar data for various applications. Our review serves as a practical reference for beginners developing mmWave-radar-based applications by utilizing machine learning techniques.

## 1. Introduction

The development of millimeter wave (mmWave) radar sensors during the past ten years has been spurred on by numerous research applications, including civilian and non-civilian applications [1,2]. With the latest improvements in chip technology and lowered cost, the mmWave radar sensor has gained widespread popularity in a wide range of applications. The mmWave radar system includes a transmitting antenna, a receiving antenna, and a signal processing system to determine an object’s dynamic information, such as range, velocity, and angle of arrival (AoA). The mmWave radar transmits a mmWave signal into space by striking an object, and this signal gets reflected. The receiving antenna captures the echo signal, which is then mixed with a transmitting signal to obtain an intermediate-frequency (IF) signal. This IF signal is processed to obtain object information. Various mmWave radars and working bands are shown in Table 1. mmWave radars operate in the frequency range between 24 GHz and 300 GHz. Processing IF signals allow for the measurement of an object’s range, velocity, and angle of arrival (AoA) [3].

The performance of signal processing is continually being improved along with hardware components. The performance of mmWave-radar-based sensing is entirely based on detecting mmWaves reflected by objects and subsequent signal processing. The performance of the mmwave radar is independent of external lighting conditions and works well even in low-light conditions and dazzling light. mmWave radar sensors are now widely used in a variety of civilian applications, including obstacle detection, motion recognition, localization, and tracking, owing to low-cost chip technology and improved reliability [4]. As a result, these improvements in radar technology and digital signal processing lead to good accuracy in range and velocity estimation and better resolution in contrast with other traditional radars. In addition to the benefits listed above, mmWave radar has superior penetration capacity through various weather conditions like rain, fog, and snow.

Today’s radar-based sensing is more diverse. Applications range from civilian to military and include a variety of automotive, industrial, and medical applications. Research in the field is being refined as a result of technological advancements and more accurate detection. To perceive the surrounding environment, mmWave radar sensors can easily be integrated with other imaging sensors. Multi-sensor fusion effectively uses data from multiple sensors to augment one another and improves the ability to extract detailed information about targets under a variety of environmental and climatic conditions [3]. The mmWave radar is more often used in various applications when compared to other sensors, such as RGB cameras, ultrasonic sensors, infrared sensors, and light detection and ranging (LiDAR). Various applications demand the fusion of one or two sensors along with mmWave radar [5].

The advantages mentioned above have increased the use and popularity of mmWave radars. This article focuses on different state-of-the-art mmWave radars with key technical specifications. The main focus of our study is on state-of-the-art mmWave radars, their key performance metrics along with suitable applications, key measurements and their interpretation, mmWave radar frequency selection criteria based on specifications and application requirements, and machine learning techniques for mmWave-radar-based sensing. Our review stands out as unique from other reviews due to its application focus on the use of machine learning methods and provides a quick review of mmWave radar models with specifications. The main significant aspects of this paper are highlighted in Table 2, presenting how it differs from earlier surveys.

The remainder of this article is organized as follows: Section 2 focuses on the performance metrics of mmWave radar sensing. Section 3 presents the measurements and interpretation of the mmWave radar sensor. Various mmWave-radar-based applications and machine learning techniques are focused on in Section 4. Finally, Section 5 concludes the article.

## 2. Performance Metrics in mmWave-Radar-Based Sensing

**Figure 1:**
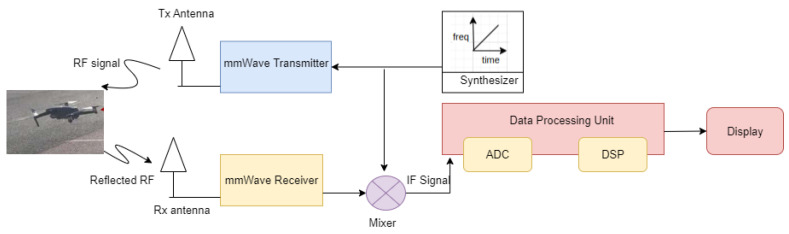
Architecture of the mmWave radar sensor.

Currently, mmWave radars have become ubiquitous, with a wide variety of applications. The types of components in mmWave radars are shown in Figure 1. The synthesizer generates the chirp, the transmitter transmits this chirp signal, the transmitted chirp is reflected off the objects in front of the radar, and the reflected signal is received via the receiver antenna. The RX signal and TX signal are mixed to generate the resultant IF signal. By processing the IF signal, all the key parameters can be estimated. When multiple objects are in front of the radar, one Tx chirp generates multiple reflected chirps from different objects, and the IF signal will have multiple tones corresponding to each of the reflections. The purpose of the analog-to-digital converter (ADC) is to digitize the IF signal. Fast Fourier transform (FFT) is performed on this digitized IF signal to obtain the range profile.

Early radar systems were primarily utilized for navigation and object detection at short ranges. They were used in maritime navigation, for example, to detect other vessels or impediments in foggy circumstances. However, advancements in semiconductor technology and processing led to advancements in the development of advanced radar systems [7]. The mmWave radar provides better antenna miniaturization than other traditional radars. Broadband radio frequency (RF) signals allow for the better resolution capability of the radar, which is the key to many high-performance automotive, medical, and industrial applications. Additionally, their enhanced portability and affordable chip have made them suitable for usage in various day-to-day applications.

A mmWave frequency-modulated continuous wave (FMCW) radar accurately estimates the range and velocity of multiple targets from the radar sensor without the need for more transceivers [9]. For the accurate estimation of the range and velocity of multiple targets, one TX antenna and one RX antenna are sufficient. However, the angle of arrival (AoA) estimation of multiple targets demands more hardware resources, such as more TX antennas and more RX antennas. The greater the number of transceiver antennas, the better the AoA estimation performance will be. However, while detecting objects using one mmWave radar, signals from other nearby radars cause interference, which eventually degrades the performance of the mmWave radar. However, there are some mitigation techniques, though it is still challenging, and this is still an ongoing research topic.

**Figure 2:**
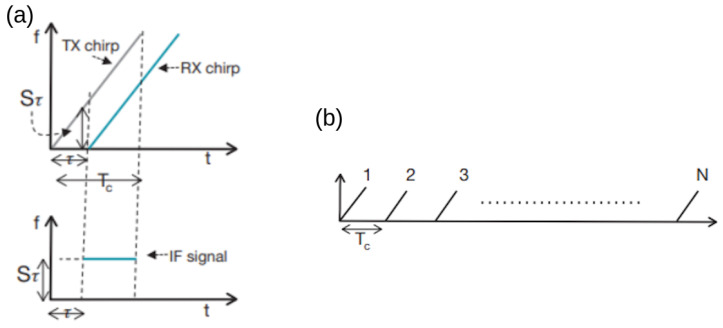
(**a**) IF signal and (**b**) chirp frame [12].

**Figure 3:**
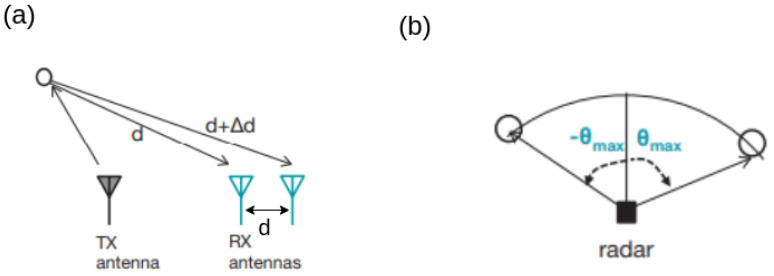
(**a**) Two antennas are required to estimate AoA and (**b**) maximum angular field of view [12].

To select the suitable mmWave radar based on the application, the following performance metrics play a crucial role [10,11]:

- *Range*—The range is estimated by analyzing the frequency content in the IF signal. As shown in Figure 2a, transmitted and received chirps as a function of time for a single object detected. It can be observed that the received chirp is a time-delay (τ) version of the transmitted chirp.
  The time delay can be measured as:
  (τ)=2R/C
  where *R* is the distance to the detected object and *C* is the speed of light.
  The initial phase of the IF signal is $\phi_{0}$
  $$(\phi_{0})=4\pi R/\lambda$$
  The range is computed as:
  $$R=C\times f_{IF}/2S$$
  The maximum range is decided via the sampling frequency of this IF signal. The larger the sampling frequency, the better the maximum range capacity of the radar will be. The other deciding factor for the maximum range is the transmission power. There is no hardware constraint on the accurate range estimation of multiple targets. One TX antenna and one RX antenna are sufficient.
  $$\mathrm{Rmax}=F_{s}C/2S$$
  Another fundamental limitation for maximum range comes from the transmitting power:
  $$Rmax={\left(\left(\sigma P_{t}G_{TX}G_{RX}\lambda^{2}T_{meas}\right)/\left(4\pi^{3}SNR_{min}kTF\right)\right)}^{\frac{1}{4}}$$
  where $P_{t}$ is output power of device; $G_{TX}$ is the TX antenna gain, $G_{RX}$ is the RX antenna fain; σ is the radar cross-section of the target (RCS); $T_{meas}$ is total measurement time; SNR is the signal-to-noise ratio; *k* is the Boltzmann constant; and *T* is antenna temperature.
- *Velocity*—mmWave radar estimates the velocity of multiple targets using the phase difference between IF signals, as illustrated in Figure 2b. There is no hardware constraint for the accurate velocity estimation of the multiple targets. One TX antenna and one RX antenna are sufficient.
  The phase difference is derived as ΔΦ = $4\pi \;VT_{c}$/λ, where $T_{c}$ is the chirp duration time. The velocity is computed as:
  $$V=\lambda \Delta \Phi /4\pi \;T_{c}$$
  The measurement is unambiguous only if |ΔΦ| < π. We can derive that vs. < λ/4$T_{c}$.
  The maximum possible velocity estimation depends on how fast chirps can be transmitted.
  $$\mathrm{Vmax}=\lambda /4T_{c}$$
- *Angle of arrival*—Estimating the angle of arrival of one object requires at least one TX antenna and two RX antennas as shown in Figure 3. The greater the number of RX antennas, the better the AoA estimation performance will be. By using MIMO, more transceiver elements can be made with a limited number of TX and RX antennas. However, accurate AoA estimation of multiple targets demands more hardware resources, such as more TX and RX antennas.
  The phase difference between the IF signals of the two receivers is derived as:
  (ΔΦ)=2πΔR/λ
  Under the assumption of a planar wavefront basic geometry Δ R = dsin(θ), where d is the distance between the receiving antennas. The angle of arrival θ is computed from Δϕ
  $$\left(\theta \right)=\sin^{-}\left(\left(\lambda \Delta \Phi \right)/\left(2\pi d\right)\right)$$
  The unambiguous measurement of the angle of arrival requires that (ΔΦ) < π.
  This leads to:
  $$\left(\theta_{max}\right)=\sin^{-}\left(\left(\lambda \right)/\left(2d\right)\right)$$
- *Range resolution*—The shortest distance at which two objects can become close while still being detected as two distinct objects via radar. The smaller this distance, the better the resolving capability of the radar will be. A radar with greater RF bandwidth gives better range resolution.
  The smallest frequency differences of an IF signal are related to the chirp duration, $T_{c}$.
  $$\left(\Delta f\right)>\frac{1}{T_{c}}$$
  where $T_{c}$ is the observation interval or chirp duration.
  $$\left(since\left(\Delta f\right)=\frac{S2\Delta R}{c}\right)$$
  $$\left(\Delta R\right)>c/2ST_{c}=c/2B\left(sinceB=ST_{c}\right)$$
  Range resolution depends only on the RF bandwidth swept by the chirp.
  Range resolution=C/(2∗B)
  where C is the speed of light, and B is the radar bandwidth. For example, a bandwidth of 4 GHz gives a range resolution of 3.75 cm.
- *Velocity resolution*—The smallest velocity difference the two targets can have while still being detected via radar as two distinct targets with two distinct velocities. Velocity resolution can be improved by increasing the frame time. A frame consists of a number of series of chirps.
  $$\left(\Delta \Phi \right)=4\pi \;VT_{c}/\lambda$$
  One can mathematically derive the velocity resolution (Vres) if the frame period $T_{f}$ = N$T_{c}$.
  V > Vres = λ/(2 $T_{f}$)
  $$\mathrm{Vres}=\lambda /(2T_{f})$$
  where $T_{f}$ is the frame time.
- *Angle of arrival resolution*—The angle of arrival resolution is the smallest angle that can be formed between two targets and radar while still being detected via the radar as two distinct targets. The smaller the angle of arrival resolution, the better the radar’s resolving capability. Extending the number of antennas (both TX and RX) improves the AoA resolution.
  $$\left(\theta \left(res\right)\right)=2/N_{RX}$$

All the performance parameters, such as range, velocity, angle of arrival, range resolution, velocity resolution, AoA resolution, maximum range, maximum velocity, and maximum AoA, are analytically tabulated in Table 3.

The following are important factors to take into account when choosing a mmWave radar [10,11]:

- *Sensor type*—The RF bandwidth, IF bandwidth, ADC sampling rate, range resolution, velocity resolution, and AoA resolution are important key factors in deciding the performance. mmWave radar sensors are broadly categorized into three sensor types: long-range, medium-range, and short-range radars, as listed in Table 4. The appropriate sensor should be chosen depending on the application demands and needs.
- *Frequency and bandwidth*—It is advantageous to have a high-frequency sensor; it uses a low antenna size and gives a better angular resolution. High bandwidth offers a high-range resolution. A 77 GHz frequency radar with a 4 GHz bandwidth gives a range resolution of 3.75 cm. Popular mmWave radar models with specifications are listed in Table 5.

- *Accessories*—As the needs of mmWave radars change, upgrading the firmware and software becomes necessary. Hence, choosing a manufacturer that can offer stable software updates is important. mmWave radars have been integrated with Lidars in [35,36] to provide better results; additionally, radars fused with cameras or infrared sensors are studied in [37,38,39]. Texas Instruments (TI) introduced a commercial radar, TDA3x, board for radar camera fusion to provide effective tracking and detection applied in [40]. There is a discussion of fusion techniques in [41,42], such as low-level and feature-level fusion.

## 3. Measurements from mmWave Radar Sensor and Interpretation of the Data

### 3.1. Range Profile

**Figure 4:**
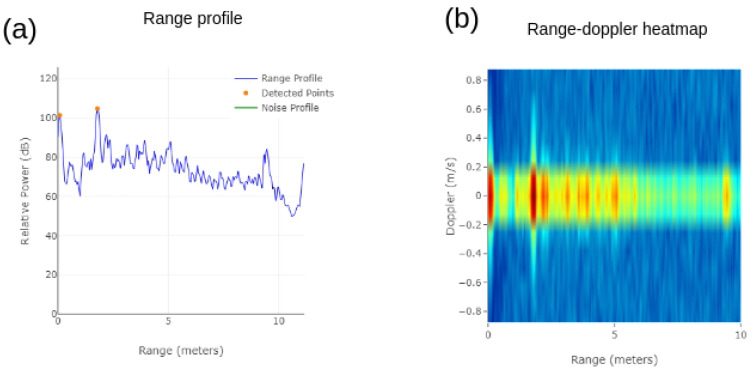
(**a**) Range profile and (**b**) range–Doppler heatmap.

A sample range profile measured using the millimeter wave radar sensor is illustrated in Figure 4a. Taking the fast Fourier transform (FFT) of an intermediate-frequency signal yields the range profile. This range profile depicts the relative reflected power from the targets as a function of range. The target is indicated via the peaks in the range profile. Targets with a large cross-section reflect more power, producing a stronger peak. Targets located near the radar produce a strong peak in the range profile.

A range–Doppler heatmap displays multiple targets with their speeds as a function of range, as shown in Figure 4b. Stationary targets have zero Doppler while moving targets have a range–Doppler map with non-zero Doppler values. This essentially provides dynamic information about the targets, such as their velocity and range.

### 3.2. Range–Azimuth Heatmap

**Figure 5:**
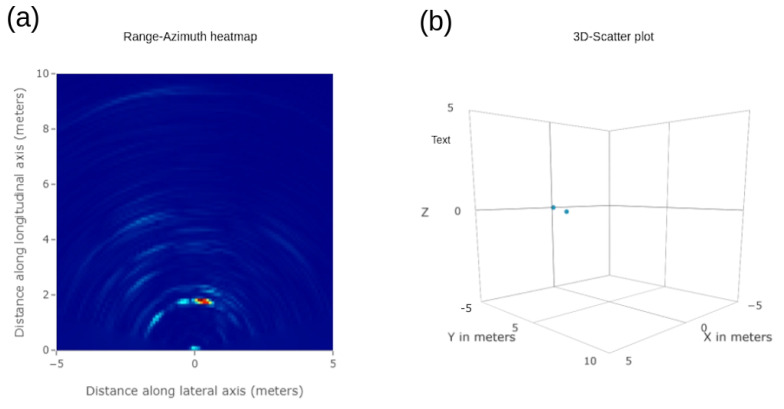
(**a**) Range–azimuth heatmap and (**b**) 3D scatter plot.

The range–azimuth heatmap in Figure 5a is intended to display radar cube information corresponding to a zero Doppler bin for every combination of range and angle bin. It essentially gives the location information about the targets with respect to radar in a Cartesian coordinate system. The colored signal power representation within the plot shows the range and angle coordinate points where the targets are located.

### 3.3. Three-dimensional Scatter Plot

The detected targets are displayed in 3D space by selecting a non-zero elevation resolution as the antenna configuration, as shown in Figure 5b.

## 4. Applications of mmWave Radars and Machine Learning Techniques

**Figure 6:**
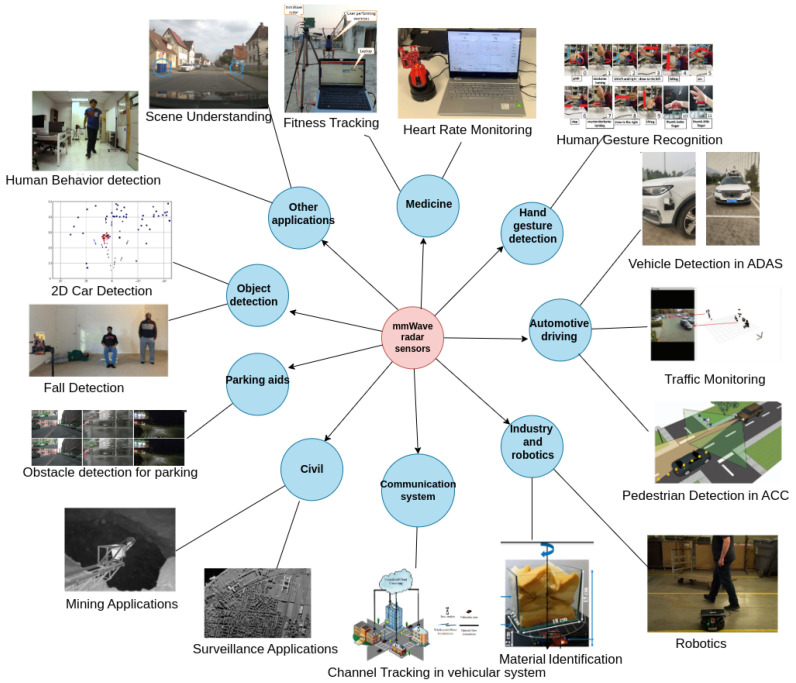
Applications of mmWave radar sensing.

mmWave radars are used in a variety of applications, as illustrated in Figure 6. These include automotive applications, industrial applications, military applications, medical applications, robotics and automation applications, civilian applications, and security and surveillance applications.

### 4.1. Automotive Applications

**Figure 7:**
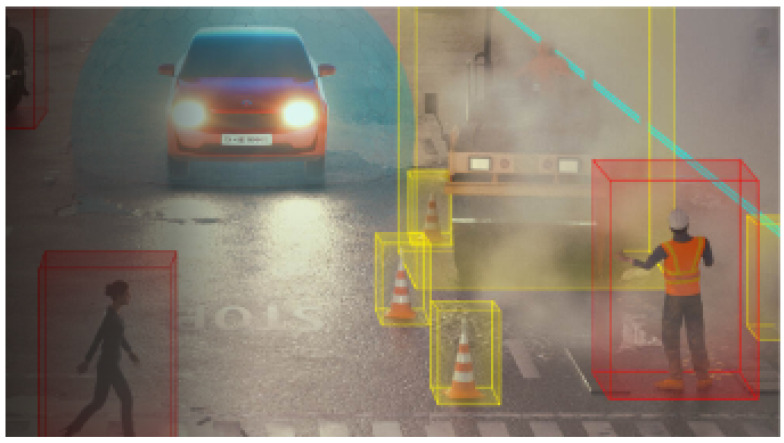
Advanced driver-assistance system [57].

Automotive applications use mmWave radar sensors to accurately localize and measure the radial range, velocity, and AoA of moving objects. The applications include adaptive cruise control, autonomous emergency braking, blind spot detection, lane change assistance, front cross-traffic alert, rear cross-traffic alert, automated parking, body/chassis applications, and in-cabin applications. The relative positioning of vehicles has been estimated using mmWave radar in [43]. Vehicle detection in advanced driving assistant systems using automotive radar with range–azimuth–Doppler dimensions is studied in [44]. A 2D car detection system for autonomous driving applications is studied in [45]. The ability of an autonomous vehicle to perceive and comprehend its surroundings is studied in [46]. The use of mmWave radars for vehicle detection in self-driving applications is studied in [47]. The use of mmWave radar sensor and vision sensor fusion for obstacle detection in autonomous driving is studied in [48]. To obtain high accuracy in new advanced driver-assistance systems (ADASs), mmWave radars have been used in vehicles [49], as shown in Figure 7. A 60 GHz mmWave radar has been used to reduce driver distractions with real-time hand gesture recognition instead of touchscreens and wearable components [50]. Machine-learning-based hand gesture recognition is studied in [51] using CNN and LSTM. People occupancy detection in a vehicle has been implemented utilizing mmWave radar [52]. Furthermore, contactless non-intrusive vehicle occupant detection is studied in [53], and autonomous navigation by predicting vehicle location utilizing mmWave radar is studied in [54]. Radar sensors placed at the front and rear corner of the car that form beams for front and rear blind spot detection, as well as for cross-traffic alert, are demonstrated in [55]. mmWave radar became a solution for ground-based traffic monitoring, and the management of both terrestrial and aerial vehicles using angle estimations in [56]. The detailed automotive applications and associated radar details are tabulated in Table 6.

### 4.2. Industrial Applications

**Figure 8:**
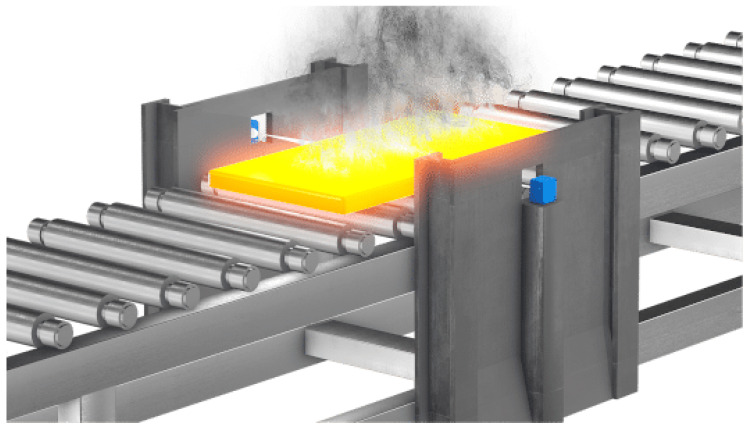
Width measurement in a cold and hot rolling mill [74].

mmWave radars have become popular as they provide precise measurements that are useful in industrial applications. Industrial applications include level sensing of fluids, volume identification for solids, infrastructure systems, surface quality assessment in production industries, and vibration monitoring. Utilizing mmWave radar for automatic crack detection to distinguish between cracked and uncracked ceramic tiles and for quality control of packaged ceramic tiles is presented in [68]. In industrial processes, to have control over the usage of liquid and identify leakages, mmWave radars have been utilized for accurately measuring fluid levels in tanks, as presented in [69]. In [70], a framework for accurate material identification for six different materials using mmWave radar is presented. In addition, the volume of the materials has been determined. Using low-power transmission signals and reflections with a non-line-of-sight (NLOS) method for detecting moving objects has been studied in [71]. This study includes a model for the echo signal of the NLOS target by considering the multipath effect and the weak target echo signal issues. The detection and classification of gases and aerosols have been implemented in [72]. Detecting the vibrational target objects by modifying shaking frequencies and assessing the performance is studied in [10]. The monitoring of the mass flow of pneumatically transported bulk materials using mmWave measuring is presented in [73]. mmWave radars are useful in metal production industries, where they require the precise measurement of slabs of copper, steel, and aluminum in production. In rolling mills, mmWave radar sensor technology is useful to provide accurate measurements, even in smoky, hot, steamy, and dusty conditions, as shown in Figure 8. The detailed industrial applications along with the radar utilized are tabulated in Table 7.

### 4.3. Medical Applications

**Figure 9:**
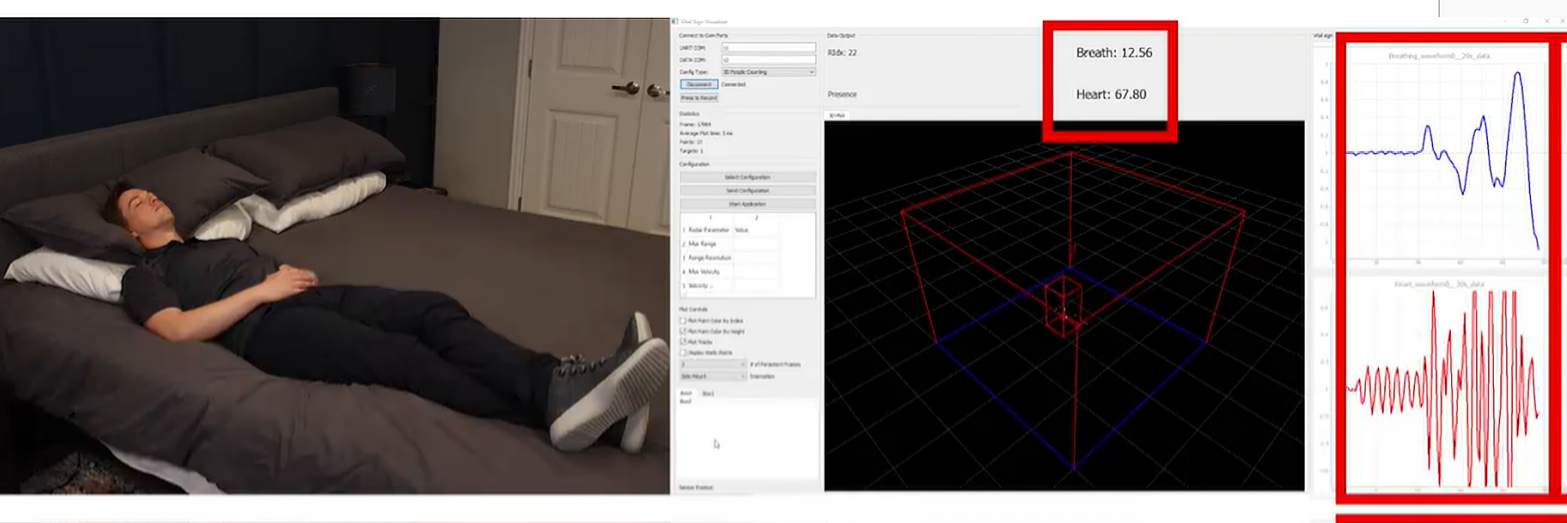
Contactless patient monitoring application [82].

Medical applications for mmWave radars have also gained importance due to their sensitive detection capability and the penetration of mmWave signals in biological tissues. Using mmWave radar, various glucose concentration levels in blood samples to distinguish the healthy or diabetic have been studied in [81]. In [82], contactless breathing rate and heart rate monitoring of patients using mmWave were implemented, as shown in Figure 9. mmWave radar sensors have been used for monitoring vital signs via non-contact means in a robust way [83]. The use of mmWave radar in real-time human motion behavior detection is presented in [84]. The recognition of multiple patient behaviors has been simultaneously studied utilizing mmWave radars in [85]. The real-time detection and tracking of human skeletal positions for patient monitoring is studied in [86]. Considering body movements as micro-motion parameters for real-time fitness tracking via non-contact means has been studied in [87]; additionally, fitness tracking by classifying and counting exercises is presented in [88]. The detection of sleeping pose identification utilizing mmWave radar has been implemented in [89]. For skin diagnosis applications, mmWave radar has been utilized in [90]. The utilization of a wearable radar sensor for continuous blood pressure monitoring is presented in [91]. Detailed medical applications utilizing mmWave radars are tabulated in Table 8.

### 4.4. Robotics and Automation Applications

**Figure 10:**
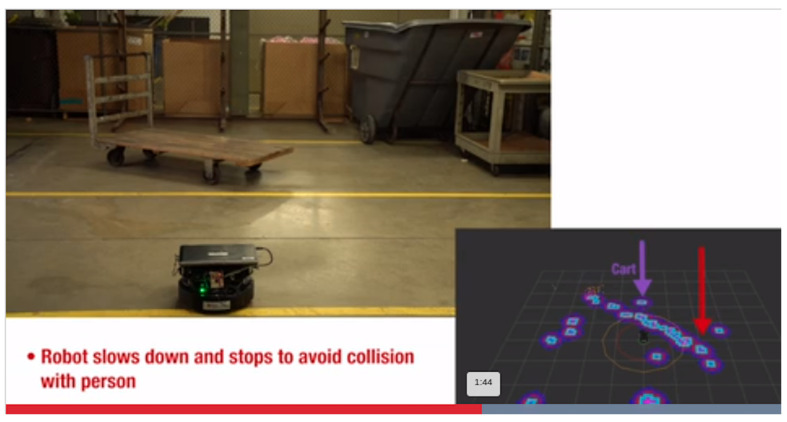
Robotics application [100].

Robotics applications include both indoor and outdoor environments. Detecting transparent objects such as glass walls is very important in autonomous navigation. mmWave radars reliably detect glass walls [99]. They are also quite reliable as ground-speed radars in agricultural and warehouse robots [99]. Using the same ground-speed radar, it is possible to sense the surface edges if the radar is mounted in front of it, facing toward the ground. Safeguards around robotic arms are another important field wherein mmWave radar plays a vital role. Mapping and navigation is another important application where mmWave radars are used in indoor environments. Robotic applications for human path tracking and collision avoidance are explored utilizing mmWave radars [100]. The detection of obstacles and avoiding collision in a 360-degree path in robotics using mmWave radars is demonstrated in [101], as shown in Figure 10. Incorporating an antenna on package sensors with a wider field of view in both azimuth and elevation helps in the intelligent sensing of transparent objects and dark objects, which is studied in [102]. Glass walls and the materials behind them can be detected using mmWave radar sensors [99]. Detailed robotics and automation applications and associated radar details are tabulated in Table 9.

### 4.5. Security and Surveillance Applications

**Figure 11:**
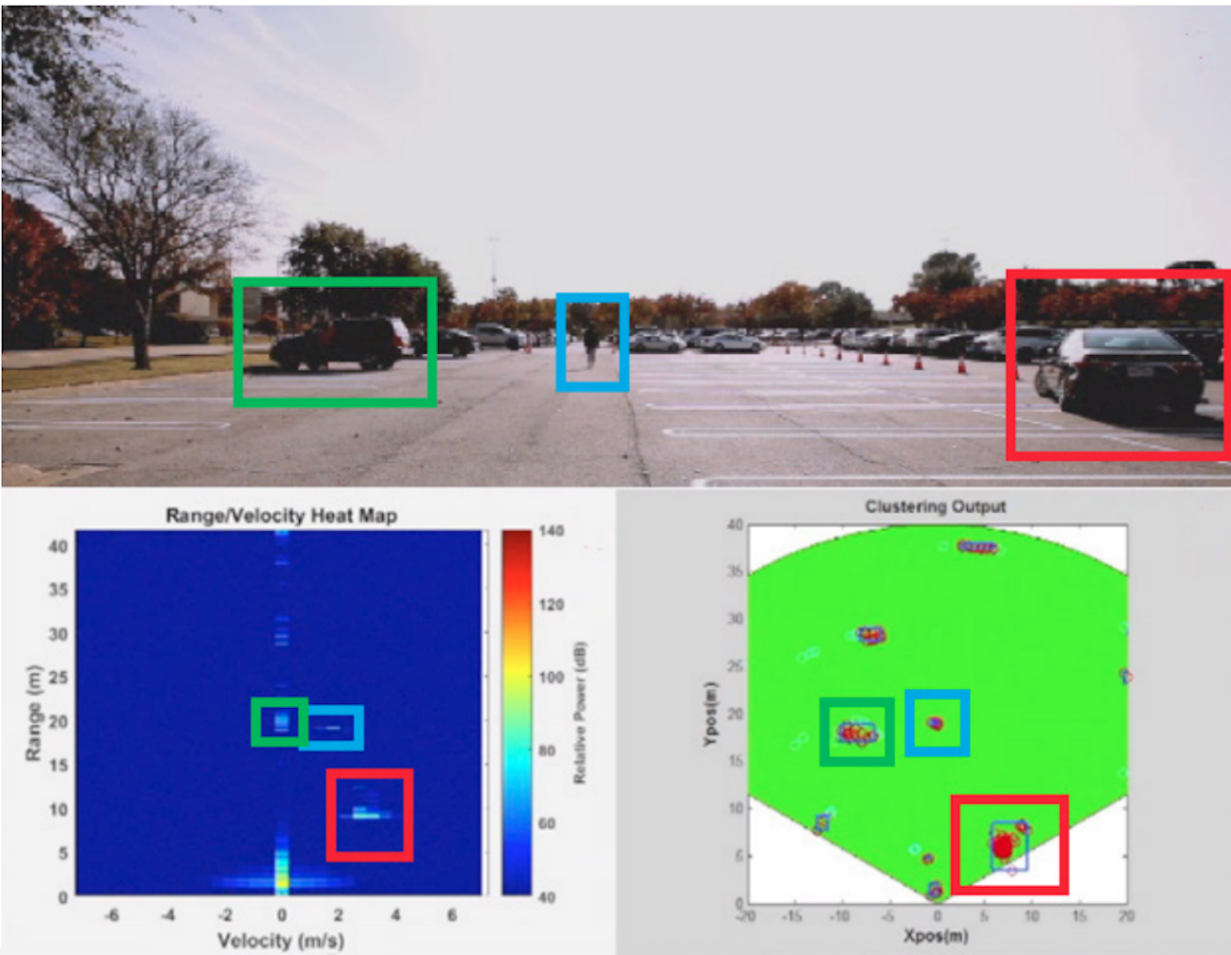
Traffic monitoring application [113].

mmWave radars are useful in security and surveillance applications because they can detect moving objects and obstacles in low-light conditions. In particular, personal screening and maintaining security aspects are discussed in [105]. mmWave radars are being used in air traffic control systems and low-altitude space surveillance applications to detect and display the position of aerial vehicles. Aerial vehicle activity monitoring with radar range and angle measurements is studied in [106]. Airborne radars for obstacle avoidance, landing aids, automotive radars for collision avoidance, and driving safety support are studied in [107]. In [108], airborne surveillance with navigational aid on the ground using mmWave synthetic aperture radar (SAR) is implemented, which tracks the actual flight route and records it. Unmanned aerial vehicle (UAV) detection with respect to a range of up to 40 m using low-cost mmWave sensors is reported in [2]. In airports and other sensitive places, mmWave radars are utilized in the identification of intrusions in [109]. Vehicle detection and tracking in traffic monitoring applications with a range greater than 100 m are shown in Figure 11. A richer radar point cloud representation for a traffic monitoring scenario is shown in [110]. People tracking using radar applications for consumers in indoor and outdoor environments is presented in [111]. Furthermore, channel tracking for a vehicular communication system is studied in [112]. Detailed security and surveillance and civilian applications and associated radar details are tabulated in Table 10.

### 4.6. Civilian Applications

**Figure 12:**
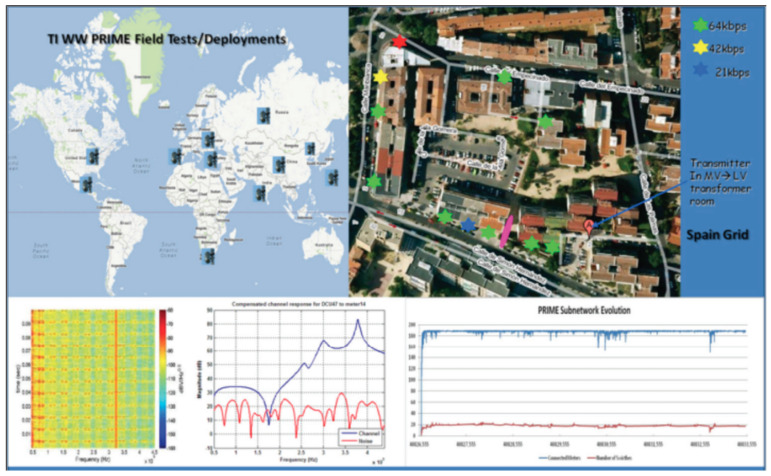
Power line communication [124].

**Figure 13:**
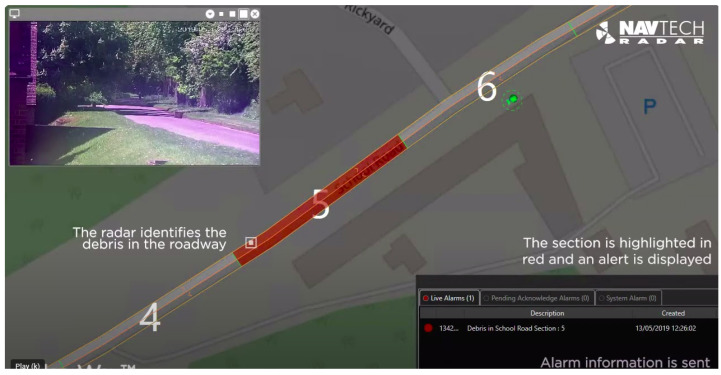
Debris detection on airport runways [125].

mmWave radars have grown in popularity because they are robust to adverse weather conditions and find many uses in the civilian sector. The creation of a drone setup by integrating a mmWave sensor to detect power lines up to a 40-m range with improved performance and fast detection is shown in Figure 12. Debris detection on airport runways, early risk warnings for helicopters, power line detection in flying paths, and malicious drone detection are some of the applications of mmWave radars. Detecting small foreign objects on airport runways for a safe landing is studied in [117], as shown in Figure 13. mmWave radars reliably detect high-voltage invisible power lines in snowy and inclement weather. Risk avoidance with early warnings from mmWave sensors to rescue helicopters is discussed in [114].

In [120], a cooperative radar sensing network is implemented for tracking small, hidden, unauthorized unmanned aerial vehicles (UAVs). Micro-UAV detection for defense applications using 24 GHz mmWave radars is presented in [126]. The classification of birds and drones using radar micro-Doppler signatures is presented in [127]. The detection and mitigation of GPS spoofing for drones is explored in [128]. Aircraft runway extraction in low-visibility conditions for a safe landing is investigated in [129]. Furthermore, in [115], the use of mmWave radars for the safe landing of helicopters in inclement weather conditions based on height drift data is studied. Another interesting application is implemented in [130], which utilizes a CNN architecture to generate radar maps for recognizing and classifying various real road images captured from gravel, mud, and river surfaces.

### 4.7. Other Applications

Object detection is one of the earliest applications of mmWave radar, which extends to human fall detection, as presented in [131]. Hand gesture recognition for user interactions with computers is studied in [132]. A model for long-range gesture recognition is investigated in [133]. Furthermore, the real-time recognition of macro-gestures is presented in [134]. Human pose estimation through occlusions and walls is explored in [135]. The implementation of mmWave harmonic sensors to track small insects is conducted in [136]. The use of 61 GHz mmWave radar for human face classification is investigated in [137]. Non-contact skin sensing for analyzing human emotional arousal and stress status using mmWave radars is implemented in [97]. The use of mmWave radar combined with GNN and LSTM for human activity recognition is explored in [138].

In addition to the aforementioned areas, mmWave radars are also used in underground mining with range measurements [139], spying on phone calls [80], efficient soil moisture sensing [140], micro-action recognition systems [141], mmWave radar and audio signal fusion for speech recognition [142]. Recent advances in sensor technology, combined with machine learning techniques, have also enabled new applications for mmWave radar sensors to be developed.

The use of mmWave radar combined with machine learning algorithms has grown in popularity in recent years. As shown in Table 11, we provide an overview of machine learning algorithms that are widely used in computer vision and related fields and have been applied to radar signal processing. The applications of machine learning include object detection, classification, clustering, and tracking, utilizing radar data. IF signals from radars contain a predefined set of target features, and these IF signals are used in machine learning models to make subsequent predictions. However, deep learning algorithms are based on multiple layers of neural networks to learn high-level feature representations from input radar IF data, which are then used to make intelligent decisions.

Furthermore, many research works are in progress that input radar signals into various deep learning techniques for object detection, such as those in [45,143,144,145]; object classification, such as those in [146,147]; object segmentation, such as those in [46,148]; and multi-class target classification, such as those in [149], using mmWave radar range–angle images. Furthermore, target classification using the range FFT of a mmWave radar’s statistical features is studied in [150]. The utilization of various deep learning techniques and micro-Doppler patterns from radar data for object classification is explored in [151]. Multi-person identification with distinct micro-Doppler signatures is studied in [152].

**Table 11:** Machine learning techniques for mmWave radar sensing.

References	Year	Method	Application	Comments
[88]	2016	CNN, data transformation techniques	Fitness tracking	Can classify different exercises with 95.53 accuracy and is capable of counting repetitive exercises. Counting repetitive exercises improves accuracy.
[132]	2016	Random forest algorithm	Hand gesture recognition	RF offers 86% per-gesture accuracy with raw data. RF with Bayesian Filter offers 92% per-gesture accuracy with raw data.
[84]	2019	Convolution neural network (CNN)	Human behavior detection	Point cloud data are processed using the CFAR algorithm. The usage of micro-Doppler information on human activities with CNN produces an accuracy above 99%.
[131]	2019	NN is compared with SVM, DT	Fall detection	Attains 98% accuracy with NN backpropagation. Evaluated on only three possible human positions with coordination points.
[153]	2019	CNN, ConvLSTM, RF	Received power prediction	Power prediction from image works effectively with rotated 3D CNN, and spatiotemporal features are predicted with a Random forest algorithm. Received power of 500ms with high accuracy and RMS errors less than 1.0 is achieved.
[112]	2019	LSTM	Channel tracking in vehicular system	Accurate user channel prediction, and less overhead rate. Usage of LSTMs is to predict the user channel based on past channel-state information.
[45]	2019	PointNets	2D car detection	Using PointNets for classification with segmentation. Mutli-class object detection needs to be investigated.
[46]	2020	CNN, RNN	Scene understanding via classification	CNN with grid maps as input for classifying static objects. RNN with point clouds as input for classifying dynamic instances.
[47]	2020	DBSCAN, Faster R-CNN	Vehicle detection	The proposed method performs better as it is a DBSCAN method based on elevation resolution and also removes noise points using filters. Using Faster R-CNN achieves 96% accuracy by representing the target with the density of the point cloud.
[110]	2020	Point clouds, GMM	Multimodal traffic monitoring	GMM performs point cloud segmentation from sensor-collected point clouds. Can extend with DBSCAN for classifying more transportation modes.
[48]	2020	SAF-FOC framework	Obstacle detection	Feature-level fusion performs well compared with data-level and decision-level fusion. To cover 360 coverage, the framework can be extended with multiple sensors.
[86]	2020	CNN	Detecting human skeletal pose	Radar data to image representation with the help of depth, azimuth, and elevation information of reflection points to identify skeletal position. Proposed an architecture with significantly reduced computational complexity with reused weights, and it also provided lower localization error, such as 3.2 cm depth and 2.7 cm elevation.
[138]	2021	Graph neural network with LSTM	Human activity recognition and gesture recognition	Iteratively extracts the point cloud features and updates the graphs. Excellent action recognition performance compared to other methods.
[154]	2021	SVM	Shape classification and object detection	Uses SVM with RFB kernel to achieve an accuracy of 96%. Comparatively less accuracy is obtained to classify multiple target objects.
[98]	2022	CNN	Automatic monitoring of heart rate and breathing rate	Obtained 87% classification accuracy by forming low, average, high, and a combination of six classes using CNN. Removes the noise caused via vibrations and gives clear rate.
[70]	2022	CNN, K-nearest neighbor	Material identification	K-NN uses two feature sets, while CNN uses the material’s distinctive features for identifying the materials. Enhanced classification accuracy of 98% in identifying the six materials at three different volume levels.

## 5. Conclusions

mmWave radar sensors have significant advantages compared to other sensors, making them an ideal solution for a vast number of applications. This article discussed the key performance parameters as well as the interpretation of radar measurements for mmWave radar sensors. The most recent mmWave radar advances and cutting-edge mmWave radars were thoroughly reviewed. The use of mmWave radar sensors was discussed in a variety of applications, such as automotive, industrial, robotics and automation, medical, security, and surveillance fields, as well as others. Finally, machine learning techniques applied to mmWave radar sensor data were investigated.

The future of mmWave radar technology seems promising, with plenty of room for growth and expansion. Here, we present some major trends and developments to keep an eye on in future years. mmWave radars are projected to play a critical role in advanced driver-assistance systems (ADASs) and driverless vehicles in automotive applications. In the future, the increased integration of mmWave radar sensors in automobiles is likely to improve safety, enable autonomous driving, and improve situational awareness in a variety of weather conditions. mmWave radars have the potential to transform industrial applications, such as non-destructive testing, quality control, and process automation. Future advancements may result in smaller, more adaptable, and cost-effective industrial mmWave radar systems. There is significant interest in employing mmWave radar for medical applications, such as the remote monitoring of vital signs, fall detection for the elderly, and early illness identification in healthcare and medical imaging. Healthcare-related mmWave radar equipment may advance in the future. mmWave radar systems are useful in security and surveillance applications, such as perimeter monitoring, intrusion detection, and surveillance in complex settings. Future advancements could result in more complex and integrated security solutions. In the field of IoT and smart cities, mmWave radar sensors could find uses in smart cities for traffic control, environmental monitoring, and public safety. Space exploration uses mmWave radar technology for remote sensing, landing, and planetary exploration. As space exploration advances, mmWave radar devices may play an important part in future space missions. Continued advances in signal processing methods and the application of machine learning techniques will enhance the capabilities of mmWave radar systems, allowing for better object detection, tracking, and imaging. The miniaturization of mmWave radar components and their integration into smaller and more diversified devices may be future trends, making them more accessible for a larger range of applications. Challenges include mitigating interference from other mmWave sources, dealing with atmospheric effects, and ensuring regulatory compliance.

## Figures and Tables

**Table 1:** Operational frequencies within the bandwidth spectrum.

Working Band (GHz)	Bandwidth (GHz)	Resolution
24	0.25 GHz	60 cm
77	4 GHz	3.75 cm
60	7 GHz	2.1 cm
94	2 GHz	7.5 cm
100	9 GHz	1.6 cm
300	40 GHz	0.375 cm
300	20 GHz	0.75 cm

**Table 2:** Comparison of our work with earlier review articles.

Reference	Year	Focus Area	SP	MI	Appl	PM	ML/DL Tech.	Comments
[6]	2011	Different mmWave technology specifications	✗	✗	✓	✗	✗	Restricted to limited technologies and data processing
[7]	2017	Focus on architecture, estimation techniques	✓	✓	✓	✓	✗	Restricted to advanced driver assistance system
[4]	2019	Review of digital modulation and interference mitigation methods	✓	✓	✓	✗	✗	Restricted to automated driving technology
[5]	2020	Working principles of vision sensors and performance parameters for autonomous systems	✓	✗	✗	✓	✓	Restricted to autonomous systems
[8]	2021	Uses deep learning and fusion models	✓	✗	✓	✓	✓	Deep fusion operations and datasets need to be improved
[3]	2022	To determine working principles, data representation methods, and challenges	✓	✗	✓	✓	✗	Restricted to detection applications
[1]	2022	On various on-board sensors, hardware components, software environments, and machine learning algorithms	✗	✓	✗	✓	✓	Restricted to unmanned aerial vehicle applications
**Our Work**	**2022**	**Performance metrics of mmWave radar, state-of-the-art mmWave radar models available on the market, radar data interpretation, applications of mmWave radars, machine learning techniques for mmWave radar**	✓	✓	✓	✓	✓	**Considers sensing applications using mmWave radar sensor in broad areas of science and engineering**

**SP**—signal processing; **MI**—measurement interpretation; **Appl**—applications; **PM**—performance metrics; **ML/DL Tech.**—machine learning or deep learning techniques.

**Table 3:** Performance metrics of mmWave radar.

Range	Velocity	Angle of arrival
R = ( *C* × $f_{IF}$)/2S	V = λ ΔΦ/$4\pi \;T_{c}$	θ = $\sin^{-}$ ((λ ΔΦ )/(2π d))
**Range resolution**	**Velocity resolution**	**Angle of arrival resolution**
dres = C/2B	Vres = λ/(2 $T_{f}$)	θres = 2/$N_{RX}$
**Max range**	**Max velocity**	**Max angle of arrival**
dmax = $F_{s}$C/2S	Vmax = λ/4 $T_{c}$	θmax = $\sin^{-}$ ((λ/2 d))

**R**—target’s range from the radar; **V**—target’s Velocity; **θ**—target’s angle of arrival; **C**—speed of light; **S**—slope of chirp, B/$T_{c}$; **Δϕ**—phase difference between IF signals; **Tf**—frame time, $NT_{C}$; **B**—chirp RF bandwidth; **$f_{IF}$**—IF signal frequency; **$F_{s}$**—sampling rate of the IF signal; **$T_{c}$**—chirp duration time; **λ**—wavelength of the chirp signal; **N**—number of chirps; **$N_{RX}$**—number of receiving antennas; **d**—distance between receiving antennas.

**Table 4:** Suitable mmWave radar types with respect to the applications.

Type	Range (m)	Bandwidth (MHz)	Azimuth View Angle (deg.)	Elevation View Angle (deg.)	Applications
Long-range radar	10–250	600	±15	±5	Autonomous cruise control
Medium-range radar	1–100	600	±40	±5	Lane change assistance system, collision mitigation
Short-range radar	1–30	4000	±80	±10	Blind spot detection, parking assistance

**Table 5:** Popular mmWave radar models and their specifications.

Reference	Model Name	Range (m)	Working Band (GHz)	Azimuth Field of View (deg)	Elevation Field of View (deg)	Chip Memory (MB)	User Interface and Connectivity
[13]	TI-IWR6843AOPEVM	180	60 GHz–64GHz	±120	±120	1.75	TMMWAVEICBOOST, DCA1000, I2C, LVDS, QSPI, SPI, UART
[14]	TI-IWR1843	180	76 GHz–81 GHz	100	±15	2	CAN, LVDS, QSPI, I2C, SPI, UART, CSI-2
[15]	Delphi ESR	174	76 GHz	±10	±45	-	CAN
[16]	NavTech CIR204-h	200	76 GHz–77 GHz	360	-	-	1 Gbps Ethernet
[17]	TI-AWRL6432	250	57 GHz–64 GHz	±18	-	1	QSPI, PWM, I2C
[18]	BoschLRR3	250	77 GHz	30	-	-	CAN
[19]	Continental ARS408-21	250	77 GHz	±9	14	-	CAN 500 kbps
[20]	Continental SRR600	>180	76 GHz–81 GHz	±90	±40	-	Ethernet, CAN-FD
[21]	TI-AWR1843AOPEVM	150	76 GHz–81 GHz	140	140	2	DCA1000EVM, CAN, CAN-FD
[22]	TI-AWR1642	100	76 GHz–81 GHz	±60	±10	1.5	CAN, CAN-FD, SPI, I2C, UART
[23]	TI-IWR1642	150	76 GHz–77 GHz	±100	±15	1.5	CAN, CAN-FD, QSPI
[24]	NXP-TEF810X	250	76 GHz–81 GHz	±18	-	1.5	LVDS, CSI2
[25]	NXP-SAF85xx	250	76 GHz–81 GHz	±18	-	5.5	SGMII Ethernet, dual CAN FD
[26]	NXP-TEF82xx	250	76 GHz–81 GHz	±18	-	0.576	CSI-2, LVDS
[27]	Continental-ARS540	300	76 GHz–81 GHz	±60	±60	1	CAN, Ethernet
[23]	TI-IWR1443	60	77 GHz–79 GHz	±65	±15	1.5	LVDS, DCAN, QSPI, CSI2
[28]	TI-AWR1443	60	76 GHz–81 GHz	±65	±15	1.5	LVDS, DCAN, QSPI
[29]	TI-IWRL6432	60	57 GHz–64 GHz	±65	±15	1	I2C, SPI, UART, QSPI
[30]	Continental-ARS4-A	250	77 GHz	±75	±20	1	CAN, Ethernet
[31]	TI-AWR2243BOOST	150	76 GHz–81 GHz	±90	±90	1.5	SPI, UART, I2C, CAN-FD
[32]	TI-AWR1243	250	76 GHz–81 GHz	±60	±15	1	SPI, MIPI-CSI2, UART
[33]	NXP4D-S32R45	300	77 GHz	±56	±56	1	PCIe, Ethernet, CAN-FD
[34]	RDK-S32R274	200	79–81 GHz	±30	±30	1	Ethernet, CAN-FD

**Table 6:** Automotive applications using popular radars.

Reference	Year	Application	Radar Used
[58]	1997	Intelligent cruise control with collision warning	FMCW (76 GHz–77 GHz)
[59]	2017	Blind spot detection and warning system	AWR1843 (76 GHz–77 GHz)
[60]	2017	Automated emergency breaking	TI-AWR1243 (76 GHz–78 GHz)
[60]	2017	In-car occupant detection	TI-AWR1642 (76 GHz–81 GHz)
[61]	2017	Driver vital sign monitoring	TI-AWR1642 (77 GHz)
[62]	2018	Automotive body and chassis sensing applications	TI-AWR1642 (77 GHz)
[50]	2018	In-car controlling with gestures	FMCW-mmWave (60 GHz)
[63]	2019	Automated parking system	TI-AWR1843 (77 GHz)
[64]	2020	Lane change assistance with obstacle detection	TI-AWR1843AOPEVM (77 GHz)
[65]	2020	Parking assistance with obstacle detection	TI-AWR1642BOOST (77 GHz–81 GHz)
[66]	2020	Debris detection for automotive radar	mmWave (76 GHz–81 GHz)
[67]	2021	Automotive vehicle detection in parking lot	TI AWR2243BOOST-MIMO (76 GHz–81 GHz)
[65]	2022	Motor cycle safety and Blind spot detection	TI-AWR1843AOP (76 GHz–81 GHz)
[55]	2022	Automotive corner radar for cross traffic alert	TI-AWR1843EVM (76 GHz–81 GHz)

**Table 7:** Industrial applications using popular radars.

Reference	Year	Application	Radar Used
[75]	2006	Surface sensing	mmWave sensor (29.72 GHz–37.7 GHz)
[76]	2013	Measuring the liquid level and interface sensing	mmWave Doppler sensor (77 GHz)
[68]	2015	Crack detection in ceramic tiles	V-Band Imaging Radar (60 GHz)
[69]	2017	Fluid level sensing	TI-IWR1443 (77 GHz)
[77]	2018	Material classification	FMCW radr with Infineon’s DEMO-BGT60TR24 sensor (60 GHz)
[78]	2018	Motion detection and intersection monitoring	IWR6843 60 GHz radar
[79]	2019	Foam detection in chemical applications	IC with mmWave ssensor (80 GHz)
[10]	2020	Obtaining the performance on detecting vibrational targets	FMCW 80 GHz sensor integrated on SiGe chip
[80]	2022	Eavesdropping and spying on phone calls	TI-AWR1843BOOST (77 GHz)
[70]	2022	Material identification	TI-IWR1443 FMCW (77 GHz–81 GHz)

**Table 8:** Medical applications with popular radars.

Reference	Year	Application	Radar Used
[81]	2018	Blood glucose level detection	FMCW-XENSIV (60 GHz)
[85]	2019	Multiple patients behavior detection	TI-AWR1642BOOST (77 GHz)
[90]	2020	Skin cancer detection	Designed sensor (77 GHz)
[87]	2021	Contactless fitness tracking	TI-IWR1642 (77 GHz–81 GHz)
[82]	2022	Contactless monitoring of patients and elderly people alone	IWR6843AOPEVM (60 GHz–64 GHz)
[92]	2022	Measuring systolic blood pressure	TI-IWR6843AOP (60 GHz–64 GHz)
[93]	2022	Vital sign measuring	TI-IWR1443 (77 GHz–81 GHz)
[94]	2022	Health monitoring with posture estimation	TI-IWR6843 (60 GHz–64 GHz)
[95]	2022	Blood pressure monitoring	TI-AWR1843 (77 GHz–81 GHz)
[96]	2022	Cardiorespiratory rate monitoring	Commercial FMCW (122 GHz)
[97]	2022	Galvanic skin test to assess mental acuity and stress levels	TI-AWR1843 (77 GHz)
[98]	2022	Automated heart rate and breathing rate monitoring	TI-AWR1443BOOST (77 GHz)

**Table 9:** Robotics and automation applications with popular radars.

Reference	Year	Application	Radar Used
[102]	2019	Intelligent robot for transparent object sensing	IWR6843 (60 GHz)
[103]	2020	Robot-mounted mmWave radar for tracking heart rate	IWR6843 (62 GHz)
[54]	2020	Predicting autonomous robot navigation	FMCW (77 GHz)
[101]	2020	Collision detection and avoidance	IWR6843 (60 GHz)
[104]	2020	mmWave radars as safe guard robots	IWR6843 (60 GHz)
[100]	2021	Automated indoor navigation and path tracking	AWR6843 (77 GHz)
[99]	2021	Glass wall and partition detection	IWR1443BOOSTEVM (77 GHz)

**Table 10:** Security and surveillance and civilian applications with popular radars.

Reference	Year	Application	Radar Used
[114]	2006	Power line prediction in helicopter rescue	mmWave radar (94 GHz)
[115]	2008	mmWave radars for safe helicopter landing	Radar module with 94 GHz
[116]	2010	Providing indoor security of short range	mmWave SAR (77 GHz)
[117]	2010	Debris detection on airport runways	mmWave radar (73 GHz–80 GHz)
[118]	2013	Concealed threat detection	W-band (75 GHz–110 GHz)
[108]	2015	Surveillance imaging applications	MIRANDA radar (35 GHz and 94 GHz)
[113]	2018	Traffic monitoring	IWR1642EVM 77 GHz radar
[119]	2019	Human target detection, classification, tracking	ISM band (24 GHz MIMIC)
[120]	2020	Tracking of malicious and hidden drones	mmWave (77 GHz)
[121]	2021	Ego-motion estimating in indoor environments	TI-AWR1843BOOST (76 GHz–81 GHz)
[2]	2021	Unmanned aircraft system detection and localization	AWR1843 Boost (76 GHz–81 GHz)
[106]	2021	Aerial vehicle locating and air traffic management	AWR1843 (76 GHz–79 GHz)
[122]	2021	3D human skeletal pose estimation	TI-AWR1843 (77 GHz)
[123]	2023	Indoor positioning system	IWR6843ISK (60 GHz–64 GHz)

## Data Availability

Data sharing not applicable.
